# CD44, a marker of cancer stem cells, is positively correlated with PD-L1 expression and immune cells infiltration in lung adenocarcinoma

**DOI:** 10.1186/s12935-020-01671-4

**Published:** 2020-12-07

**Authors:** Chenyue Zhang, Hui Wang, Xia Wang, Chenglong Zhao, Haiyong Wang

**Affiliations:** 1 Department of Integrated Therapy, Fudan University Shanghai Cancer Center, Shanghai Medical College, Shanghai, 200032 China; 2 Department of Thoracic Surgery, Shandong Provincial Hospital Affiliated to Shandong First Medical University, 324 Jingwu Road, Jinan, Shandong PR China; 3 Department of Internal Medicine Oncology, Shandong Cancer Hospital and Institute, Shandong First Medical University and Shandong Academy of Medical Sciences, Number 440, Ji Yan Road, Jinan, 250117 China; 4 Department of Pathology, Shandong Cancer Hospital and Institute, Shandong First Medical University and Shandong Academy of Medical Sciences, Jinan, 250117 Shandong P.R. China

**Keywords:** Cancer stem cell, CD44, Programmed cell death ligand-1, Lung adenocarcinoma

## Abstract

**Background:**

PD-L1 inhibitors is widely applied in lung adenocarcinoma patients. Tumor cells with high PD-L1 expression could trigger immune evasion. Cancer stem cells (CSCs) can evade from immunesurveillance due to their immunomodulating effects. However, the correlation between CSC and PD-L1 and some immune-related markers is seldom reported in patients with lung adenocarcinoma. Therefore, we aimed to ascertain their association in lung adenocarcinoma patients.

**Methods:**

We assessed CD44 expression and its association with PD-L1 in lung adenocarcinoma, using Tumor Immune Estimation Resource (TIMER), which was further validated in our patient cohort. The immune cells infiltration was depicted by CIBERSORT using GEO database. The correlation between CD44 and immune cells was also analyzed. We further evaluated the prognostic role of CD44 in patients with lung adenocarcinoma both using Kaplan–Meier plotter and validated in our patient cohort.

**Results:**

Positive association between CD44 and PD-L1 were found in lung adenocarcinoma patients. T cells CD4 memory resting cells and mast cells resting cells varied significantly between patients with CD44 high and those with CD44 low. Furthermore, positive association could be found between CD44 expression and immune cells. Arm-level depletion of CD44 was linked with B cell, CD4^+^ T cell, neutrophil and dendritic cell infiltration. Patients with higher CD44 levels had worsened overall survival (OS).

**Conclusions:**

In summary, these results demonstrate that CD44 was associated with PD-L1 and infiltration of immune cells, and was a negative prognostic factor for predicting worsened OS in lung adenocarcinoma.

## Background

Lung cancer is the most lethal cancer worldwide with a 5-year survival rate of 15% [1]. Although adenocarcinoma (ADC) accounts for a large proportion of lung cancer, most ADC patients developed metastasis upon initial diagnosis, resulting in poor prognosis. Immunotherapy, aiming to enhance the host antitumor immune defense, has rendered prolonged survival in ADC [2].

Programmed cell death ligand-1 (PD-L1) is expressed in many solid tumors including lung cancer. Interaction between programmed cell death-1 (PD-1) and its ligands could attenuate immune response via evasion of immune elimination. It has been reported that PD-L1 is overexpressed in lung adenocarcinoma patients and is associated with dismal prognosis. Therefore, immune checkpoint inhibitors (ICIs) have been widely applied in lung adenocarcinoma for its function in the blockade of PD-L1 [3–6].

Cancer stem cells (CSCs) represent an exclusive cohort of long-lived cells with their capacity to generate cellular progeny throughout life and fuel tumor growth [7, 8]. Their existence may account for the ineffective response of many therapies including ICIs, leading to poor prognosis and recurrence among patients with different cancer types. CSCs reside in a niche where immune cells, microvesicles and cytokines could stimulate their self-renewal and promote metastasis [9]. These microvesicles and cytokines might also exert immunosuppressive functions. Studies have found that CSCs can weaken T cell differentiation, proliferation and anti-tumor effects.

However, studies on CSC’s association with PD-L1 and the surrounding microenvironment in lung adenocarcinoma are rare documented. CD44 has been identified as a crucial surface marker indicating CSC in lung cancer. Lung cancer cells expressing CD44 have been reported to be enriched for stem-like properties [10].

Therefore, in the present study, we explored the association between CD44 and PD-L1 in lung adenocarcinoma. Moreover, we assessed some of the vital immune cells surrounding CSC in lung adenocarcinoma using Tumor Immune Estimation Resource (TIMER) and further validated in our patient cohort.

## Methods

### Bioinformatics analysis of CD44 expression and its association with PD-L1 and immune cells

For evaluation of CD44 expression among all cancer types, data collected from Tumor Immune Estimation Resource (TIMER) were collected [11]. For determination of CD44′s correlation with PD-L1 mRNA in lung adenocarcinoma, TIMER was also employed [11]. CD44 expression was measured in immunological subgroups of lung adenocarcinoma and drugs targeting CD44 were depicted as referenced by an integrated repository portal for tumor-immune system interactions [12]. OS between lung adenocarcinoma patients with CD44 high and CD44 low was calculated using Kaplan–Meier plotter [13].

### Patients and samples

A total of 76 patients diagnosed with lung adenocarcinoma from December, 2012 to October 2013 in Shandong Provincial Hospital affiliated to Shandong University were included in this study. All these patients underwent lung cancer radical resection, and formalin-fixed paraffin-embedded (FFPE) specimens were collected from patients. Detailed information on age, gender, smoking habits, tumor stage and lymph node stage were all documented. Scoring of CD44 expression in tumor from each lung adenocarcinoma patient was confirmed by pathologists. Tumor staging was determined according to the International Association for the Study of Lung Cancer (IASLC). This study was approved by Shandong Cancer Hospital. All included patients in this study offered written informed consent. Overall survival (OS) was defined as the interval between diagnosis and death or between diagnosis and the last observation point. For surviving patients, the data were censored at the last follow-up.

### Immunohistochemistry staining

Immunohistochemistry protocols were performed as described previously [14]. Briefly, tumor sections were stained with rabbit anti-CD44 (abcam, ab216647), rabbit anti-PD-L1 (ARIGO, ARG57681). The Goat anti-rabbit antibody was used as the secondary antibody.

### Scoring of the staining

Immunostaining of CD44 and PD-L1 was analyzed under light microscopy at 400× magnification by two independent pathologists who were blinded to the clinical data of each patient. For evaluation of CD44, the staining was graded as either high expression or low according to the median value. X-tile software was used to determine the optimal cut-off value according to the user’s manual and previous studies.

### Statistical analysis

The chi-squared test was performed to assess the association between CD44, PD-L1 and clinical pathologic features of patients with lung adenocarcinoma. Pearson’s correlation test was used to analyze the correlation between CSC marker CD44 and tumor infiltrating immune cells (B cell, CD8^+^ T cell, CD4^+^ T cell, macrophage, neutrophil and dendritic cells). OS was defined from the date of surgery to the date of death or last follow-up. To compare OS between patients in different groups, we have used Kaplan Meier analysis. And to estimate the difference in survival, we have adopted log-rank test. Univariate and multivariate analysis was also conducted using the Cox proportional hazards regression model. All the analyses were performed using SPSS 17.0 software. A p value less than 0.05 was considered statistically significant.

## Results

### Patients’ characteristics

A total of 76 patients with lung adenocarcinoma were included in the study. Among them, seventeen patients were ≥ 65 years at diagnosis. Most of the patients were non-smokers, accounting for 67.1%. The majority of patients were female (59.2%).

There were 27 patients with at T1 stage, 41 patients with T2, 4 patients with T3 stage and 4 patients with T4 stage. A total of 48 patients were without lymph node metastasis (N0), 7 patients were at N1 stage and 21 patients were at N2 stage. We also classified patients into subgroups with CD44 high and low. A total of 58 patients were CD44 low accounting for 76.3% and 18 patients were CD44 high accounting for 23.7%. Detailed information was demonstrated in Table 1.

**Table 1:** Clinical information for lung adenocarcinoma patients (N = 76)

Variables	Number	%
Age		
< 65	59	77.6
≥ 65	17	22.4
Smoke		
Yes	25	32.9
No	51	67.1
Sex		
Female	45	59.2
Male	31	40.8
T stage		
T1	27	35.5
T2	41	53.9
T3	4	5.3
T4	4	5.3
N stage		
N0	48	63.2
N1	7	9.2
N2	21	27.6
CD44		
Low	58	76.3
High	18	23.7

### CD44 is positively associated with PD-L1 expression in lung adenocarcinoma

**Fig. 1:**
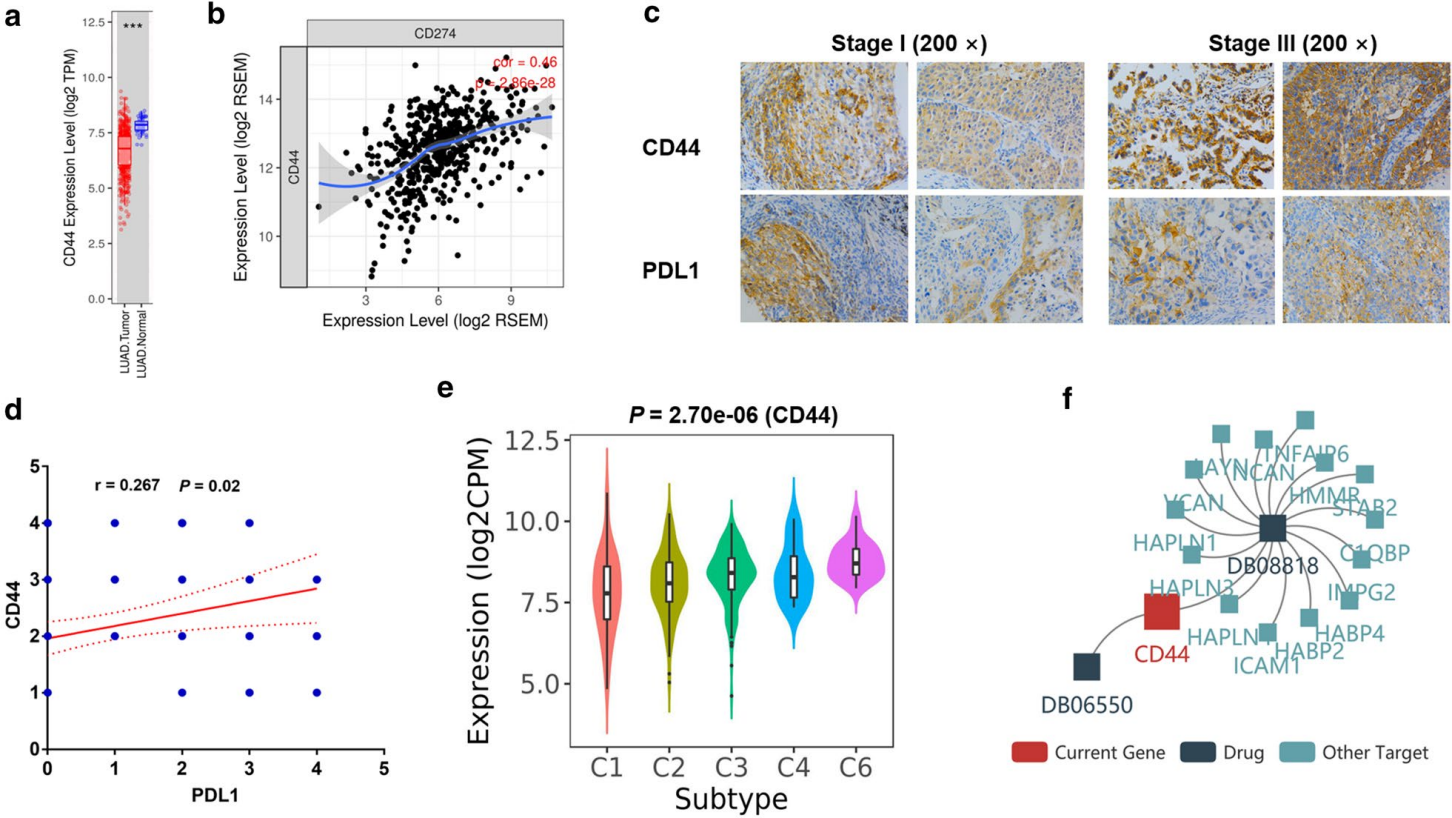
CD44 expression was lower in tumor compared with nontumor tissues and was positively correlated with PD-L1 in lung adenocarcinoma patients**. a** Lower expression of CD44 was found in tumors as compared with non-tumor tissues in patients with lung adenocarcinoma. **b** Positive correlation was found between CD44 and PD-L1(CD274) mRNA expressions using Tumor Immune Estimation Resource (TIMER). **c** Representative images were shown demonstrating CD44 and PD-L1 expression in tumors from lung adenocarcinoma patients with stage I and stage III. **d** Positive association was found between CD44 and PD-L1 in tumors from lung adenocarcinoma patients, as evaluated by immunohistology. **e** CD44 expression was analyzed in lung adenocarcinoma of different immunological subtypes. **f** Diagrams demonstrating anti-tumor drugs targeting CD44

We further examined the expression of CD44 both in tumor and nontumor tissues in lung adenocarcinoma patients. Figure 1a has demonstrated that CD44 expression was lower in tumor tissues, as compared with the non-tumor tissues in lung adenocarcinoma, using Tumor Immune Estimation Resource (TIMER). To further ascertain the impact of tumor stage on CD44 expression, we analyzed CD44 expression in lung adenocarcinoma patients with stage I and stage II/III. Results have shown that no significant difference was found between patients with stage I and stage II/III (Additional file 1: Figure S1). We further tested the correlation between CD44 and PD-L1 (CD274) mRNA expression using TIMER again. Figure 1b have shown that CD44 level was in positive proportion with PD-L1 mRNA expression (r = 0.46, *P* = 2.86e−28). To validate the correlation between CD44 and PD-L1 in lung adenocarcinoma patients with different stages, we presented representative images using tumor tissues from lung adenocarcinoma patients with stage I and stage III by immunohistochemistry. Figure 1c demonstrated that CD44 was positively correlated with PD-L1 both in stage I and stage III lung adenocarcinoma patients. Figure 1d has also demonstrated the positive association between CD44 and PD-L1. It is known that lung adenocarcinoma could be divided into several immunological subtypes, as exemplified by wound healing, IFN-gamma dominant, inflammatory, lymphocyte-depleted, immunologically quiet and TGF-beta dominant. CD44 level in these subgroups were demonstrated in Fig. 1e, which varied significantly in these subgroups (*P* = 2.70e−06). Currently, drugs targeting CD44 have been under investigation and development. For instance, one study has shown that DB08818 and DB06550 are representative drugs targeting at CD44, as depicted in Fig. 1f.

### CD44 is associated with infiltration of immune cells

**Fig. 2:**
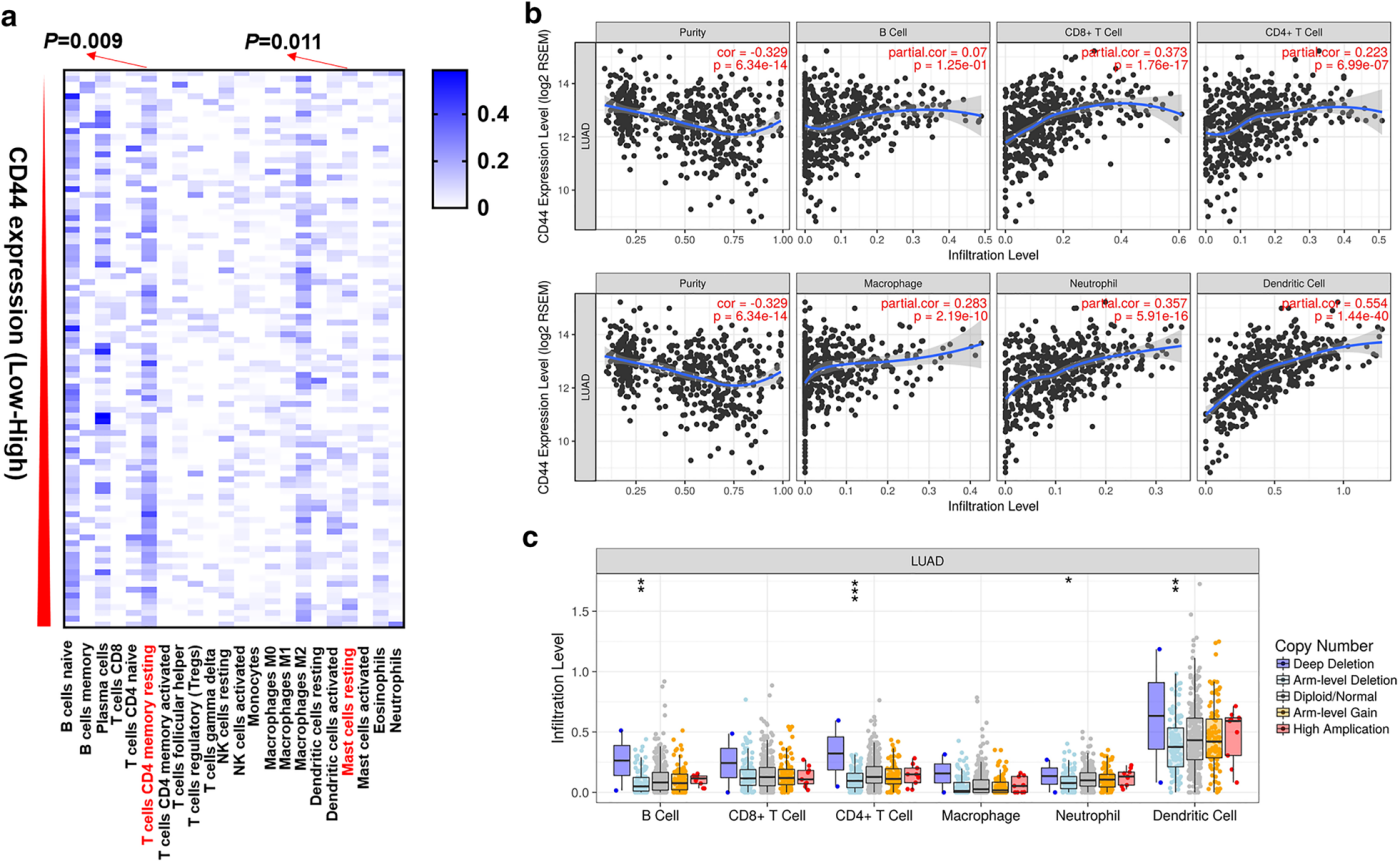
CD44 was positively associated with immune cell infiltration. **a** Landscape of immune cell infiltration in CD44-high and CD44-low lung adenocarcinoma patients, using GSE103584 database. **b** The association between CD44 and infiltration of immune cells (B cells, CD8 + T cells, CD4 + T cells, macrophage, neutrophil, dendritic cells) using Tumor Immune Estimation Resource (TIMER). **c** The association between CD44 copy number variation (deep deletion, arm-level deletion, diploid/normal, arm-level gain, high amplification) and the infiltration of immune cells (B cell, CD8 + T cell, CD4 + T cells, macrophage, neutrophil, dendritic cells) was analyzed. ***: < 0.001, **: 0.001 ≤ ** < 0.01, *: 0.01 ≤ * < 0.05

Since the association between CD44 and PD-L1 was confirmed in lung adenocarcinoma patients, we then tested the landscape of immune cells in lung adenocarcinoma patients using GSE103584. Results have shown that a cohort of immune cells existed in lung adenocarcinoma, such as B cells naïve, B cells memory, plasma cells, T cells CD8, T cells CD4 naïve, T cells CD4 memory resting, T cells CD4 memory activated, et al. Among these immune cells, we have found that in CD44 high and CD44 low lung adenocarcinoma, there was a significant difference in the amount of T cells CD4 memory resting and mast cells resting (Fig. 2a). To determine the association between CD44 and immune cells, particularly those immune cells which could boost immunity, TIMER was employed. Results have found that CD44 was positively correlated with B cell (r = 0.07, *P* = 1.25e−01), CD8+ T cell (r = 0.373, *P* = 1.76e−17), CD4+ T cell (r = 0.223, *P* = 6.99e−07), macrophage (r = 0.283, *P* = 2.19e−10), neutrophil (r = 0.357, *P* = 5.91e−16) and dendritic cell (r = 0.554, P = 1.44e−40) (Fig. 2b). We further analyzed the association between various forms of CD44 copy number and immune cells B cell, CD8+ T cell, CD4+ T cell, macrophage, neutrophil and dendritic cell infiltration in lung adenocarcinoma. Results have shown that CD44 arm-level depletion was correlated with the immersion of B cell, CD4+ T cell, neutrophil and dendritic cell (Fig. 2c).

### CD44 could serve as a prognostic biomarker for OS in lung adenocarcinoma

**Fig. 3:**
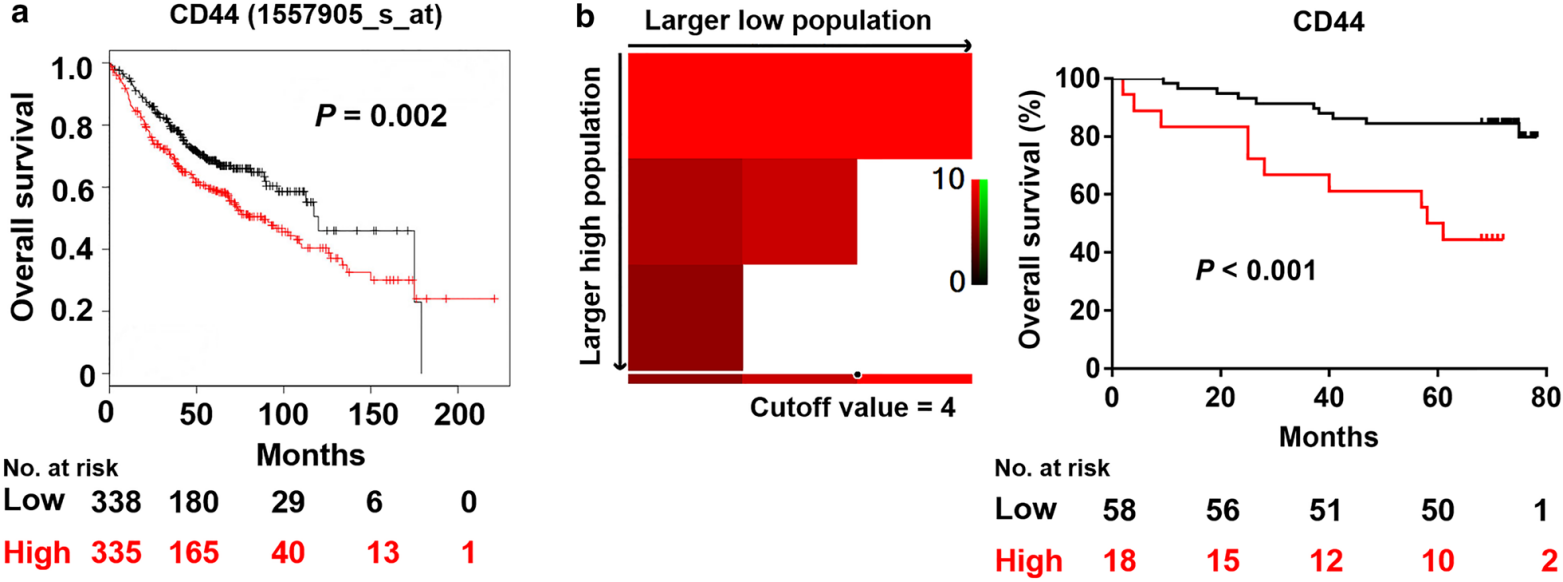
CD44 serves as a valuable predictive marker in lung adenocarcinoma patients. **a** The role of CD44 in affecting overall survival (OS) was analyzed using Kaplan Meier plotter and number of patients at risk at different times was demonstrated. **b** Cut-off value was determined using X-tile model. The role of CD44 in affecting OS was evaluated in our patients with lung adenocarcinoma

To analyze the role of CD44 in affecting OS in patients with lung adenocarcinoma, Kaplan–Meier plotter was used. Results have shown that lung adenocarcinoma patients with low CD44 expression had significantly prolonged OS than those with high expression (*P* = 0.002) (Fig. 3a). Furthermore, specimens of lung adenocarcinoma patients were collected from Shandong Provincial Hospital. We used X-tile software to calculate the most efficient cut-off value of CD44 expression that could most significantly distinguish the outcome of patients with lung adenocarcinoma. OS was further analyzed using Kaplan Meier analysis (Fig. 3b). The result also showed that lung adenocarcinoma patients with relatively low CD44 expression had improved OS compared with those with high expression (*P* < 0.001). In univariate analyses, sex, T stage, N stage and CD44 were found to be independent predictors for OS (*P* = 0.049, *P* = 0.002, *P* < 0.001, *P* = 0.001 respectively). By multivariate analyses, N stage and CD44 were proven to be independent predictors for OS (*P* = 0.024 and *P* = 0.022 respectively) (Table 2). Together, these data suggest that increased CD44 expression was correlated with the poor OS among lung adenocarcinoma patients, and the CD44 expression could serve as a useful biomarker for predicting prognosis among patients with lung adenocarcinoma.

**Table 2:** Univariate and multivariate analyses of prognostic factors in lung adenocarcinoma patients (N = 76)

Variables	Univariate analysis		Multivariate analysis	
	Wald χ2	*P*	HR (95%CI)	*P*
Age	0.084	0.771		NI
< 65				
≥ 65				
Smoke	1.787	0.181		NI
Yes				
No				
Sex	3.878	0.049		0.112
Female			Reference	
Male			2.177 (0.834–5.680)	0.112
T stage	9.563	0.002		0.369
T1			Reference	
T2			6.799 (0.766–60.343)	0.085
T3			8.541 (0.713–102.27)	0.090
T4			4.852 (0.279–84.320)	0.278
N stage	14.628	< 0.001		0.024
N0			Reference	
N1			3.579 (0.882–14.522)	0.074
N2			5.110 (1.577–16.560)	0.007
CD44	10.751	0.001		0.022
Low			Reference	
High			3.534 (1.197–10.435)	0.022

## Discussion

In the present study, we have found lower expression of CD44, a surface marker of CSC, in lung adenocarcinoma. Moreover, we analyzed the association between PD-L1 and CD44 in lung adenocarcinoma. Indeed, there have been studies investigating the association between PD-L1 and CSC in a multitude of cancers. Upregulated PD- L1 expression were reported to be found in breast and colon CSCs [15]. One study showed significant correlation between PD-L1 expression and CSC markers Oct4A, Nanog and BMI1 in a large breast cancer data set. It has also shown that two of the CSC markers, Oct4A and Nanog, could be induced by ectopic PD-L1 expression whereas downregulation of PD-L1 expression led to attenuated self-renewal ability of breast CSCs [16]. Another study has shown that PD-L1 expression was correlated with CSC markers and in chemoresistant colorectal cancer specimens [17]. One study led by Dong also revealed that stemness of tumor cells could be boosted by PD-L1 [18]. CD44 is involved in multiple signaling functions, cell proliferation, apoptosis, survival, migration and differentiation. Studies have also determined the functional role of CD44 in cytokine production and secretion and deemed it a typical surface marker in lung adenocarcinoma [19]. In the present study, we have found that CD44 was positively correlated with PD-L1 expression (r = 0.46, *P* = 2.86e−28) in lung adenocarcinoma using TIMER database. Their positive association was further validated in tumor specimens among patients with lung adenocarcinoma (r = 0.267, *P* = 0.02). Our finding on the association between PD-L1 and CSC was consistent with these previous reports.

In the current study, we further analyzed the microenvironment surrounded by CSC. To this end, we classified lung adenocarcinoma into six discrepant subtypes, namely wound healing, IFN-gamma dominant, inflammatory, lymphocyte depleted, immunologically quiet and TGF-beta dominant. Each of the six subtypes represents a specific microenvironment. The niche where CSCs reside is quite complex. It contains a collection of heterogeneous cells, which may result in different subtypes in one cancer [20–22]. Tumor with the same type may be inconsistent in immune-competence among different individuals. Immuno-competence, to some extent, partially reflects the microenvironment in which the tumor is involved. As our results clearly demonstrated, CD44 expressions were relatively higher in C3 (inflammatory), C4 (lymphocyte depleted) and C6 (TGF-beta dominant) subtypes.

Since tumor microenvironment is composed of large amounts of immune cells, we next detect CSC’s association with a bunch of immune cells. In the current study, B cell, CD8^+^ T cell, CD4^+^ T cell, macrophage, neutrophil and dendritic cell (DC) were all analyzed for their correlation with CSC, as reflected by CD44, in lung adenocarcinoma. B cells positively modulate immune responses and inflammation through antibody production and to promote T-cell activation and proliferation through antigen presentation. CD4^+^ T cells have been reported to contribute to the microenvironment remodeling required for sustained tumor regression. It is known that neutrophils in tumors often predict worsened outcomes. Dendritic cells (DCs) are reported to be responsible for the balance between CD8^+^ T cell immunity and tolerance to tumor antigen. Tumor-associated macrophages (TAM) can enhance tumor cell invasion and metastasis [23–27]. There have been studies reporting the interaction between CSCs and immunes cells, as mediated by growth factors and cytokines [28, 29]. For example, one study recently demonstrated that TAM could create a niche suitable for CSCs. One study has proven that tumor associated macrophages (TAMs) increase the number, tumorigenicity and drug resistance in CSCs through STAT3 activation. In turn, CSCs induce M2 phenotype in TAMs and block anti-tumor CD8^+^ responses during chemotherapeutic treatment [30]. One study showed that CSC-pulsed DCs induced the antigen-specific TH1 immune response [31].

In this study, we have found that CD44 expression was correlated with T cells CD4 memory resting and mast cells resting, using GEO database GSE103584. Moreover, in the analysis of TIMER, we have detected positive association between immune cells (B cell, CD8 + T cell, CD4 + T cell, macrophage, neutrophil and dendritic cell) with CSC, as detected and manifested by CD44.

Finally, we analyzed the role of CD44 in affecting OS in lung adenocarcinoma patients. Kaplan Meier analysis demonstrated that CD44 as a negative role in affecting OS and Cox regression analysis proved it served as a negative prognostic factor. Interestingly, although lower CD44 expression was found in tumor compared with nontumor tissues, lung adenocarcinoma patients with higher CD44 expression were found to have worsened survival. This contradiction could be attributed to the following reason: The fact that CD44 is upregulated in tumor tissues compared with non-tumor tissues may be indicative the role of CD44 in the occurrence of LUAD. However, the fact that CD44 is correlated with poor prognosis suggested that it is more linked with cancer progression and metastasis. Besides, it has to be noted that although CD44 was linked with increased infiltration of immune cells whereas found to be a negative biomarker associated with worsened OS in lung adenocarcinoma patients. We assume the following reasons may be possible: (1) Despite the infiltration of numerous immune cells surrounding CSC in lung adenocarcinoma, these immune cells do not necessarily exert anti-tumor activities. For instance, tumor associated neutrophils and TAM exert pro-tumor effects that would boost the malignant properties. Their pro-tumor effect outweighed the anti-tumor effect, which would lead to increased malignancy. (2) Since CSC was associated with PD-L1 accumulation in lung adenocarcinoma in the present study, the inhibition of anti-tumor effect exerted by PD-L1 could possibly blunt the suppressive function of immune cells on tumor cells. Studies have view PD-1/PD-L1 axis as a major pathway exerting immune-inhibitory effect, triggering a suppressive microenvironment that protects cancer cells from immune destruction [32]. Moreover, Schatton et al. reported that CSCs could downregulate T cell activation [33], which may also serve as one of the explanations for CD44′s negative role in predicting OS in lung adenocarcinoma. Previous studies have found that some unique compositions from exosomes from the different cancer stages can be employed as vehicles to judge cancer development, progression, and metastasis [34, 35]. Although the difference in CD44 expression was not observed in lung adenocarcinoma patients with stage I and stage II/III, it also holds promise that CD44 would also serve as a predictive marker for survival in lung adenocarcinoma patients.

We have first conducted the study to ascertain the association between CD44 and PD-L1 in lung adenocarcinoma. In addition, we tested the infiltration of immune cells surrounding CSC. Furthermore, the role of CD44 in predicting OS in patients with lung adenocarcinoma was also analyzed. We have demonstrated that CD44 is positively correlated with PD-L1 expression, immune cells infiltration and serves as a negative prognostic biomarker in lung adenocarcinoma. We therefore speculate that lung adenocarcinoma patients with higher CD44 expression may be surrounded by robust immune cell infiltration whereas these surrounding cells do not exert proper anti-tumor effects against CSC. We propose that lung adenocarcinoma patients with higher PDL1 expressions may evade immune cells attack. Undeniably, some points could be improved in our study. For instance, T cells and macrophages could be classified more intensively to be indicative of subtypes and activation status.

Notably, our finding is of some clinical relevance. Currently, there have been many clinical trials of immunotherapeutic approaches targeting CSCs. For instance, CSC-DC vaccine, aiming to inhibit immune suppression induced by CSC, is under investigation [25].

## Conclusion

In summary, we have demonstrated that CD44 is positively correlated with PD-L1 expression, immune cells infiltration and serves as a negative prognostic biomarker in lung adenocarcinoma. We propose that lung adenocarcinoma patients with higher PDL1 expressions may evade immune cells attack. And the association between CSC, PD-L1 and immune cells warrants further studies in large cohorts, which would lead to development of more effective drugs against lung cancer.

## Supplementary information

**Additional file 1: Figure S1.** No difference in CD44 expression was found between stage I and stage III lung adenocarcinoma patients. CD44 expression was evaluated both in stage I and stage III lung adenocarcinoma patients.

## Data Availability

The data are available from the corresponding author upon reasonable request.

## Abbreviations

CSCs: Cancer stem cells
TIMER: Tumor Immune Estimation Resource
OS: Overall survival
ADC: Adenocarcinoma
PD-L1: Programmed cell death ligand-1
PD-1: Programmed cell death-1
ICIs: Immune checkpoint inhibitors
FFPE: Formalin-fixed paraffin-embedded
IASLC: International Association for the Study of Lung Cancer
