# Supplementary figures and images for: CD44, a marker of cancer stem cells, is positively correlated with PD-L1 expression and immune cells infiltration in lung adenocarcinoma
Source: Cancer Cell Int. 2020 Dec 7;20:583. doi: 10.1186/s12935-020-01671-4 (PMC7720536; doi:10.1186/s12935-020-01671-4)

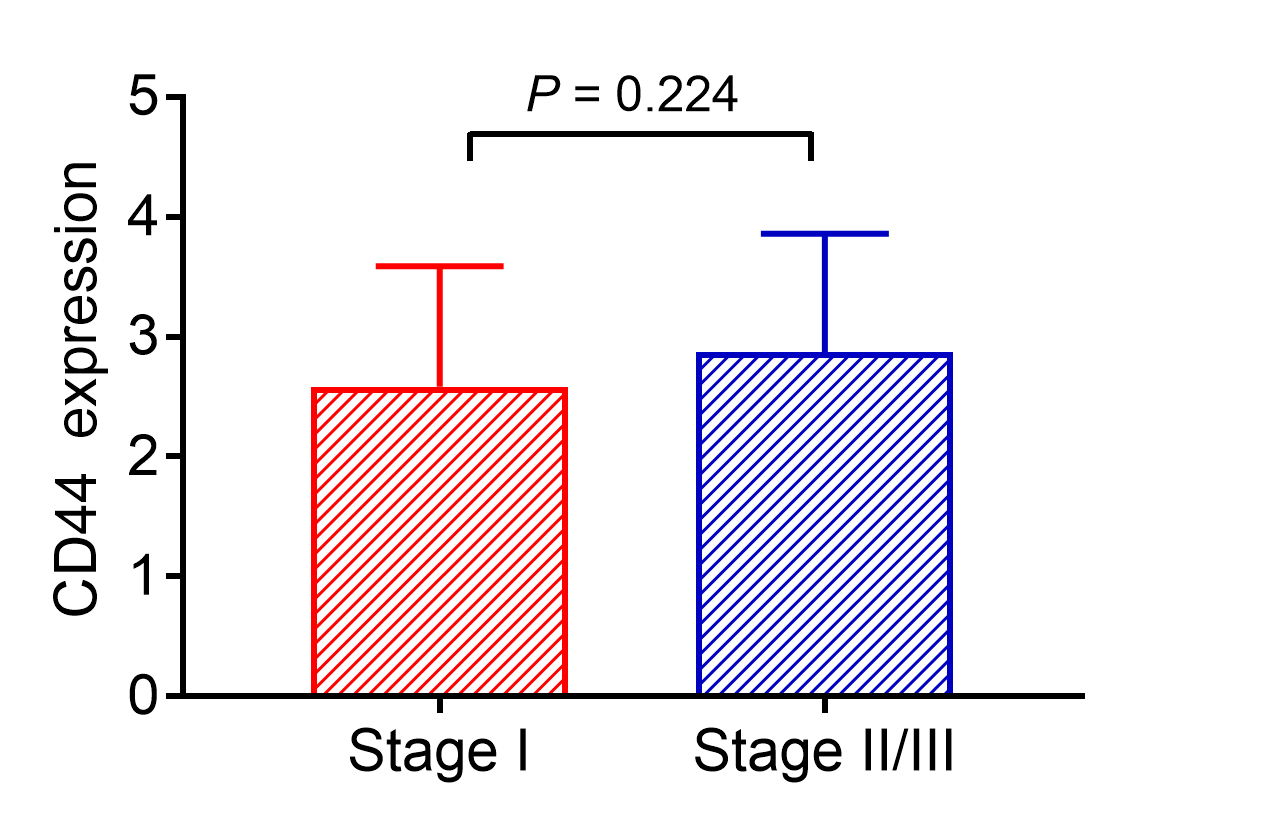

Supplement: Supplementary file 1 — Additional file 1: Figure S1. No difference in CD44 expression was found between stage I and stage III lung adenocarcinoma patients. CD44 expression was evaluated both in stage I and stage III lung adenocarcinoma patients. [file 12935_2020_1671_MOESM1_ESM.tif]
